# Experiences of moving an older parent into a care home or nursing home in the UK: a qualitative study

**DOI:** 10.1007/s12144-023-04538-9

**Published:** 2023-03-24

**Authors:** Oliver Robinson, Amy Fisher

**Affiliations:** School of Human Sciences, University of Greenwich, London, SE10 9LS UK

**Keywords:** Ageing, Care home, Life transitions, Qualitative method, Older parents

## Abstract

The study explored the personal experiences of individuals making the decision to move an older parent into a residential care facility via retrospective narrative. It aimed to gain an understanding of how individuals experienced this transition, the emotions they felt at specific moments throughout the transition, and the perceived effect it had on their psychological wellbeing. 13 semi-structured video interviews were conducted online with individuals who had been active in the decision of moving an older parent into a care home or nursing home. The data was analysed using thematic analysis, along with relational analysis to explore relations between themes. Findings informed 8 different themes, which were subsumed under three meta-themes of *The Decision Process*, *Conflicting Emotions* and *Reflective Evaluation*. It was found that the decision was recalled as a complex and often stressful negotiation between multiple stakeholders, that emotions ranged from grief to guilt and relief, and that reflections emphasised the positive that had come out of the transition. The results from this study provide valuable insights into the uniqueness of this transition from the perspective of relatives and the range of emotions experienced at different stages of the transition.

Approximately 410,000 older adults live in care or nursing homes in the UK, as of 2019 (Age, 2019; BBC News, 2020). For every one of these individuals, a complex decision must be made that typically includes adult children, other relatives and relevant professionals, to move them from a private residence to a care home or nursing home. Research within the field has identified that this entry into a care or nursing home can be a very difficult life transition, both practically and emotionally, with complex factors to consider (Dementia, 2018). This project aimed to explore the psychological transition of moving an individual into a residential care facility from the perspective of the children of the individuals involved in that a decision, through a theoretical lens based on life transitions and wellbeing.

## Theories of life transitions

According to Schlossberg (2011), a life transition occurs when major changes in external roles and life structure arise, accompanied by corresponding shifts in inner states, behaviour and relationships, leading to a period of time of change and instability. Transitions can affect physical and psychological health (Miller, 2010). Life transitions in adulthood typically involve multiple interconnected, discontinuous external changes such as changes in residence, work/study, social groups and relationship (Robinson, 2020). Bridges (2020) developed a model that focuses on the external features of life transitions. The transition process in his model starts with *endings*, during which a person leaves a social and/or physical system in which they were previously embedded, such as a residential situation, a relationship, a friendship group or a work environment. Following, and overlapping with, the endings, comes a neutral zone, in which the person or persons in transition find themselves in-between the old systems and commitments, but without a clear presence of a new beginning. For Bridges, much of the internal realignment and restructuring occurs in this liminal phase. He considers it as the seedbed for the personal growth that occurs in the *new beginnings* phase. In this phase, the transitioning person finds new roles and relationships, and a new sense of purpose. Often this phase is accompanied by renewed energy and a new sense of identity.

According to Ellis (2010), major life transitions in adult life typically take between six and twelve months. Throughout a transition, individuals are challenged to develop alternative ways to view their world or an experience, a concept known as reconstruing, which should prompt the individual to respond creatively to their new reality (Ellis, 2010). Researchers have shown that individuals can differ in their ability to cope with change, with some viewing a transition as representing great opportunities whilst others may perceive it as a loss of identity and support (Schlossberg, 1981). Whilst adulthood can be viewed as a period of continual change, with individuals facing many challenging life transitions including marriage, parenting, divorce or death of a relative (Schlossberg, 1981); moving into a residential care facility can be accompanied with many unique challenges, an aspect which will be discussed in a following section.

The internal states that life transitions lead to have the dual potential to be either disruptive and destructive, or empowering and regenerative (Almeida & Wong, 2009).This is partly due to the fact that individuals differ in their ability to cope when faced with a transition (Papadopoulous, 2020). Fisher’s (2000) personal transition theory is based around four possible pathways through transition, each of which integrate internal and external features that lead to a different outcome. The four possible pathways are *denial, disillusionment, hostility* and *moving forward*. In the denial pathway, a person experiences initial anxiety about the prospect of change, and then enters into denial about the situation, which in turn makes autonomous change through a transition unlikely. In the disillusionment pathway, the individual moves through anxiety and then into a more positive affective state about the change, but then after experiencing a further sense of threat of an uncertain future and depression at the sense of loss that comes with change, makes a decision to not follow the transition and to revert back to the pre-transition state. In the hostility pathway, the transition is followed but without a sense of acceptance or positivity. In the moving forward pathway, the ending-new beginnings transition is completed and the individual finds acceptance, purpose and happiness in the post-transition situation.

Schlossberg’s (2011) theoretical approach to coping with transition involves four elements: situation, self, support and strategies. These combine in individualised ways to predict how well an individual will cope with a transition. In this approach, the *situation* refers to an assessment of the timing, triggers and existing stressors at the time of the transition. The *self* refers to the individual’s inner ability to cope with the situation, looking at their personal characteristics and psychological resources. Resilience, optimism and ability to deal with ambiguity are all favourable characteristics which can aid an individual’s ability to cope. Next, *support* refers to the support that the individual can access throughout the transitionary period, essential to one’s sense of well-being. This support can include friendships, family and professional colleagues. The *strategies* category seeks to explore an individual’s coping responses when faced with the transition, which may include strategies to obtain more information, to positively reframe a situation or to aid the reduction of stress. Using this as a framework can be a useful aid in understanding the continuum in which individuals can cope with a transition, with various factors combining to produce a unique ability to cope for all individuals (Anderson et al., 2012).

## Factors affecting the decision for entry into a care facility

The transition of an older adult into a care home is a major life transition that follows the endings-new beginnings pathway described by Bridges (2020) as common to all transitions, and also has the complex emotional multi-outcome patterns described by Fisher (2000). Residential care facilities function to provide personal care and accommodation for individuals who need additional support in their daily lives, which can include help with dressing, washing, eating, taking medication or using the toilet (Age UK, n.d.). Whilst a residential care facility can significantly improve the quality of life for older individuals, in addition to removing excess pressure from relatives (Davies & Nolan, 2003), for many families this transition can be a challenging decision, with many negative repercussions for family members in terms of stress, anxiety and conflict (Ellis, 2010). Conflict between family members of the older adult in question is common in the transition to care, with families and older adults typically disagreeing on the nature of the decision (Clarke & Bright, 2006). This can result in intense periods of stress, misunderstandings and confusion for both the older adult and their relatives (Koenig et al., 2013). Correspondingly, the process is an emotionally difficult period time for family caregivers; they report emotions ranging from ambivalence to guilt, powerlessness, anxiety, and a sense of loss (Sanderson & Meyers, 2004; Strang et al., 2006).

Researchers have identified many factors that influence the decision to place an older adult into a residential care facility (Dellasega, 1997). Most of these transitions are precipitated by a crisis event (Merla et al., 2018). These can include an acute illness or injury that leads to admission to hospital. Castle (2003) found, in a sample of 306 older nursing home residents, that 78% of transitions to the home occurred following hospitalization. Cognitive impairment, loneliness, incontinence, wandering behaviour or increased pressure on their partner/carer are also typical drivers (Davies & Nolan, 2003; Merla et al., 2018). The decision-making process involves both the decision to transition the older adult to care, and then selecting a home (Davies, 2005).

It is common that family members involved in the process have to support both their older parents and children. This demanding situation means having to juggle many responsibilities including child-rearing, personal interests, employment and care for their older friends and relatives (Merla et al., 2018). Consequently, the admission into a care facility is regarded as rushed and pressured (Merla et al., 2018). In some circumstances, relatives may spend years caring for their relative before they are admitted into a care facility, at which point they may be perceived as visitors rather than carers; symbolising a major transition for them too (Edge, 2007). Qualitative research using focus-groups has suggested that for some family members, the transition of moving their relative into a care facility may negatively affect their mental health (Cottrell et al., 2020), as the transition can be associated with feelings of self-doubt, blame, loneliness, isolation and powerlessness; all of which have been reported by caregivers in previous qualitative research studies (Afram et al., 2015; Gaugler et al., 2014).

Previous research within this field has found that many older individuals can be very reluctant to move into a residential care facility (Dementia, 2018). This may stem from the widespread negative stigma surrounding care homes which is circulated in the media which highlight examples of poor practice and/or abuse and fail to call attention to the high-quality care and practice which exists in many care homes. Alternatively, individuals may fear they will be forgotten about once they move into a residential care facility away from their immediate family; both of which perceptions were identified in a study conducted by Gaugler et al. (2014). Resultantly, many individuals discuss feeling shame when they reflect on the decision that they had to make, knowing they had to respond in a way which went against their relative’s wishes, especially for relatives with Alzheimer’s who may have been unable to contribute to the decision (Afram et al., 2015).

## Rationale, aims and research questions

Whilst researchers have investigated the role that family members play in relocating a relative into a care facility, the majority of studies within this area have focused on the psychological experience of residents and staff. Relatively few studies have explored this transition from the perspective of family members, with those that do being conducted mainly in Australia and the USA, and none that we are aware of basing their analysis on transition theory. The most similar existing study to the current one was conducted by Koenig et al. (2013). They interviewed 22 residents and a family member about their decision to move into an ‘Assisted Living’ environment. The study was pioneering in terms of its empirical focus, but it is different in a number of key regards. It did not analyse the data through a lens that incorporated transition theory, and it did not focus specifically on the children of the older adult. Our rationale for focusing on children of these older adults is that these have been identified as the most likely source of informal caregiving other than a spouse (Chanfreau & Goisis, 2022). It was important to focus on those adult children who were actively involved in the decision to move the older parent to the care environment as research suggests the transition may have substantive emotional effects on them and that the decision itself is substantively a function of their goals, appraisals and expectations. With increasing aging populations worldwide, and the number of family caregivers increasing cross-culturally, it is important to consider the psychological impact of the transition for these caregivers. Whilst the role of caregivers in the UK, USA and Australia appears similar between countries, given the difference in care provision for older adults amongst these countries, we are of the view that empirical work from the UK is required to see if findings from alternative studies are aligned with this research.

As such, there appeared to be a clear niche for the proposed research. The rationale for interpreting the data through the lens of transition theory relates to (a) the assumption that the theory provides a fitting framework for this life transition, and (b) that a broad theory such as transition theory may allow for making meaningful connections between the transition to care homes and other kinds of transition that involve families and residential variables. The data for the study was collected before the COVID-19 pandemic, and thus represents the psychosocial situation in non-pandemic years. While we recognise that the residential care environment has been changed by COVID-19 and government responses to it, we believe that views of the dynamics of the transition into care from before the pandemic remain relevant.

The chief aim of this study was to explore and interpret the experiences of the children of an older parent moving into a nursing or care home within the UK. As noted by Merla et al. (2018), family members are typically involved in the care of older adults and the decision to move an older adult into a residential care home or nursing home. As a result, they offer a valuable perspective that has the potential to further enhance our understanding of the circumstances leading to the decision and experience of transitioning older adults into residential care facilities. The study was led by the primary research question: How do individuals experience the transition of moving a parent into a care facility? In answering this question, the time leading up to the admission, the experience of relocation, emotions and wellbeing, and subsequent life after this transition were explored retrospectively.

## Method

### Epistemology

This research project was conducted using semi-structured interviews and thematic analysis, within an interpretivist paradigm, seeking to uncover meaning and deeper implications via intensive immersion in, and interaction with, the dataset (Somekh & Lewin, 2011). Interpretivism is based upon the assumption that social reality is shaped by human experience and social contexts, thus not singular or objective. In this way, social reality is interpreted through a process of ‘sense-making’, rather than hypothesis testing (Denscombe, 2007), with a single phenomenon having the potential for multiple interpretations rather than one truth which can be assessed with a single measurement (Creswell, 2007). Furthermore, the process of interpretive analysis is holistic and contextual, with interpretations based on language and meanings from the perspectives of the participants involved in the social phenomenon under examination (Creswell & Poth, 2018). For this reason, it was a suitable meta-perspective for framing insights into individuals’ subjective experiences of the transition of moving a relative into a care or nursing home

### Sampling and participants

In total, the sample included 13 individuals; 5 men and 8 women aged 43–77. An inclusion criterion was that individuals must have played an active role within the decision-making process of placing a parent into a residential care facility. Participants were advised not to participate if they felt discussing the matter would be too distressing for themselves. Time elapsed since the transition varied from 1 year to 10 years (See Table 1). All participants were White British or White European. In terms of recruitment, convenience sampling and snowball sampling were used to obtain the sample (Bryman, 2012). Participant recruitment was conducted through advertisements on social media platforms and through personal contacts. Whilst this sampling method limits generalisation to some degree (Marshall & Rossman, 2016), it allows access to an otherwise hard-to-reach sample (Robinson, 2014). Sampling was continued until data saturation was reached (Vasileiou et al., 2018). Data saturation refers to the point in which no new information emerges in the data analysis process and thus indicates that data collected is sufficient (Faulkner & Trotter, 2017). After 13 participants were recruited, analyse was conducted, and the analysts reviewed whether further data collection was necessary to ensure themes were fully developed. The decision was made that themes were sufficiently ‘saturated’ with content to move forward to write-up and reporting.

**Table 1:** Sample information

Participant pseudonym	Age	Occupation	Years since the transition	Relationship to older person	Transition of mother of father?	Cognitive / physical status	Residential status of parent
Maggie	55	Catering assistant	10 years	Daughter	Mother	Dementia	Independently
Jennie	60	Retired – economics teacher	6 years	Daughter	Father	None reported	Independently
Peter	56	NHS governance support officer	8 years	Son	Father	Dementia	Independently
Annie	58	Interior designer	5 years	Daughter	Mother	Dementia	Independently
Patricia	55	Teacher	7 years	Daughter	Mother	Parkinson’s	With relative
Patrick	77	Retired – former banker	5 years	Son	Mother	None reported	Independently
Jessica	69	Retired – art designer	3 years	Daughter	Mother & Father	Dementia – mother None reported - father	Independently
Carol	43	Play specialist	1 year	Daughter	Mother	Parkinson’s	Independently
Mark	52	Retired – insurance broker	1 year	Son	Father	Alzheimer’s	Independently
Janet	54	Health setting client manager	2 years	Daughter	Mother	Alzheimer’s	With relative
Malcolm	54	Civil engineer	10 years	Son	Mother	Dementia	Independently
Gloria	57	Curriculum team manager	6 years	Daughter	Mother	Dementia	Independently
Josh	43	Manager of sports club	1 year	Son	Father	None reported	Independently

### Data collection

Semi-structured, in-depth interviews were used to collect the data between May and July 2020 (Marshall & Rossman, 2016). Interviews were conducted after receiving ethical approval from the University of Greenwich’s School of Human Sciences Research Ethics Panel. Informed consent was obtained from all participants before commencing the process of data collection, with all participants fully debriefed after the interview and provided with links to support services. All interviews were conducted by one individual (second author).

An interview guide was used as a flexible framework to ask questions regarding the time leading up to the admission, the admission itself, and life after the admission, with a focus on wellbeing throughout. Semi-structured interviewing allowed participants to discuss the key topics they felt were important without any imposed limitations, with probes used throughout to encourage participants to expand on their comments and elaborate their opinions. Due to the Coronavirus global pandemic, interviews were conducted online via a video call. The interviews lasted between 25 min and 1 h, based on the participants’ narrative.

### Data analysis

All interviews were audio-recorded to assist the process of analysis, advisable when using an interpretivist approach to ensure that accurate accounts are correctly reported (Denscombe, 2007). Thematic analysis was used to elicit common themes from the interview data (Braun & Clarke, 2006). Relational analysis, which acts as a bolt-on technique to thematic analysis, was then used to explore relationships between themes and to develop an integrated model (Robinson, 2011). For Braun and Clarke, qualitative research is a process concerned with meaning and meaning-making, whilst simultaneously viewing these as context-bound. As such, thematic analysis focuses on interpretation, rather than discovery of truth, and is the product of prolonged data immersion, and theory-led reflection (Braun & Clarke, 2019).

Braun and Clarke’s (2006) approach to thematic analysis was followed. This is made up of six phases, including data familiarisation, producing preliminary codes and looking for themes. This is followed by a process of modifying the themes, naming the themes and forming the final report. Throughout the analysis, a process of abductive reasoning was used, which involved using the interview data to produce the most likely explanation whilst acknowledging that the data set was likely to be incomplete (Lipscomb, 2012).

To begin the process of thematic analysis, meaningful segments of data from each of the transcripts, which were related to the research aim and questions, were selected to create initial codes. These codes were then collapsed and synthesised into themes, accumulating in a list of quotes related to each theme. These themes were then reviewed and refined multiple times to create themes which best captured the aims of the research study. Following this method of thematic analysis encouraged the use of a systematic and rigorous approach to theme development whilst simultaneously providing flexibility (Braun & Clarke, 2019).

## Results

**Fig. 1:**
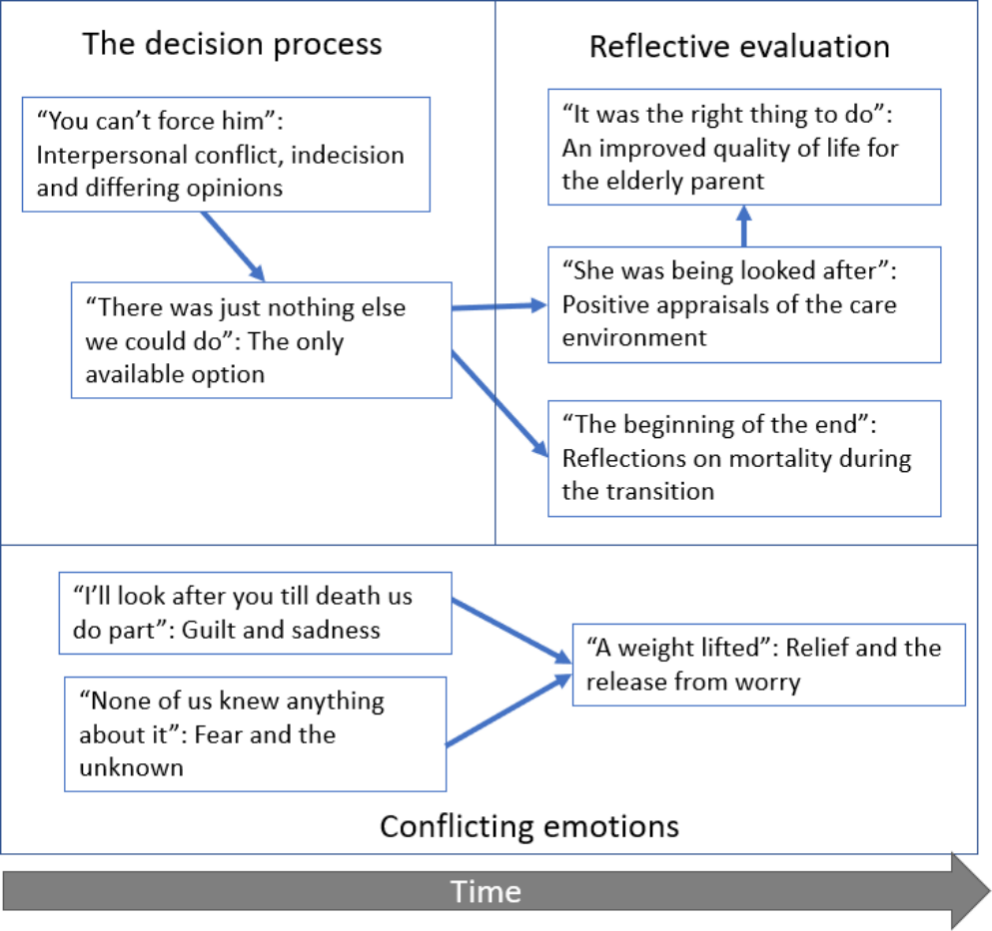
Thematic model, showing the three meta-themes and eight sub-themes with arrows showing approximate temporal relationships between themes

Eight key themes emerged through the thematic analysis, which were categorised into three higher-order themes; *The decision process, Reflective evaluation* and *Conflicting emotions*. Figure 1 provides a visual model of these themes, which is grounded in the data and the relationships between thematic categories provided by participants in their interview narratives. The arrows represent the temporal relations inferred between themes from the participant narratives, which will be discussed further in the Discussion. These temporal relations were inferred from the description of the narrative sequence of the transitional process, in which participants described the chronological flow of events and experiences, as part of the relational analysis process. We discuss this further in the Discussion section. The model conveys key features of the transition unfolding over time within the participants’ narratives. Quotes presented below are illustrative, to help describe and illuminate the content of each theme, but not exhaustive. Readers can access the full primary dataset of transcripts and analysis documents from the authors.

**Table 2:** Themes and meta-themes reported, listed by each participant

Meta-themes	The Decision Process		Reflective Evaluation			Conflicting Emotions		
**Sub-themes**	‘You can’t force him”: Interpersonal conflict, indecision and differing opinions	“There was just nothing we could do”: The only available option	“It was the right thing to do”: An improved quality of life for the older parent	“She was being looked after”: Positive appraisals of the care environment	“The beginning of the end”: Reflections on mortality during the transition	“I’ll look after you till death us do part”: Guilt and sadness	“None of us knew anything about it”: Fear and the unknown	“A weight lifted”: Relief and the release from worry
**Maggie**		X	X	X	X	X	X	X
**Jennie**		X	X		X			X
**Peter**		X		X	X	X	X	X
**Annie**					X	X		X
**Patricia**		X	X	X	X	X	X	
**Patrick**	X	X	X	X				X
**Jessica**	X	X					X	X
**Carol**	X	X				X		X
**Mark**	X		X	X			X	
**Janet**		X	X			X		X
**Malcolm**	X	X		X		X	X	X
**Gloria**		X				X	X	
**Josh**	X	X		X	X	X		X

### The decision process

#### “You can’t force him”: Interpersonal conflict, indecision and differing opinions

This theme emerged in six of the 13 interviews – it subsumes participants describing struggles with their relatives and with the older adult, when discussing the imminent prospect of the transition. These struggles were linked to the situational factors of the time leading up to the admission, including the medical needs of the older adult, their ability to contribute to the decision, and the desire / resistance of the older adult to move.

Conflicts with family members were commonly cited in this phase as a source of stress and a key challenge to overcome. For example, Jessica and her brother had opposing views about the move. She said that “it was stressful trying to find them a place and battling against my brother who we knew for financial reason didn’t want them to move out… so that was really stressful”.

5 participants spoke of their relative’s desire to continue living in their home, and were adamant that a care facility would not be appropriate for them. For example, Josh said “but he was insisting he didn’t want to move out, we [Josh and his brother] raised the issue and he was like “no no no I’m fine yeah because he is stubborn yeah” we both wanted him to move out the house many years ago… but we couldn’t as you can’t force him… if he doesn’t want to do it he doesn’t want to do it.”. Also, Mark said “my sisters and I … well all of us had floated the idea to her before that, but she firmly resisted us …”, while Carol also said that “she was adamant she didn’t want to go into a care home … she really didn’t”.

Overall, the transition to the care environment for these participants was fuelled by stressors and conflict amongst relatives, which made the decision element of the transition more complex and emotionally fraught.

#### “There was just nothing else we could do”: the only available option

The most prevalent theme that emerged, mentioned by 11 out of 13 participants, surrounded the move into a care facility being perceived as the only option available. For these 11, the transition into a care home was regarded as the final step, after they had “tried everything else” (Janet).

Peter explained how “it became obvious that really … the only place for him was a home … you know I couldn’t live with him, we couldn’t live there as a family … we couldn’t give him the proper professional help, you know he needed proper clinicians around him to care for him … so a nursing home was the only option really, as sad as it is”. Josh also spoke of the move to a care home as being a necessary option, explaining that whilst “nobody wants to end up in a home do they, but there’s just nothing else we could do … I couldn’t … I couldn’t do anything about it because I live over here, he didn’t want to come back”.

An absence of alternative options conveys a situation where the decision was ultimately one that was about perceived forced necessity, for medical purposes, safety or quality of life. Some participants spoke of their initial aim to manage the situation themselves at home, either through taking on caring responsibilities themselves or through the provision of professional home carers. After a period of time, they recalled they came to the realisation this was no longer viable, with some commenting they had noticed their relative was not eating properly or was struggling with a lower standard of personal hygiene or medical care. Consequently, some individuals concluded they were “not really coping” (Patricia) and thus had to “make the best decision as possible for that person” (Jennie).

### Conflicting emotions

#### “I’ll look after you till death us do part”: guilt and sadness

Guilt was an emotion which was conveyed by nine participants and sadness was described by four participants. Guilt was described as the result of having transgressed an internalised ethical standard to continue looking after the older parent no matter what, or to keep them in their own home to the end. For example, Carol said that “I end up feeling guilty that you can’t do more, especially if she doesn’t think she wants to be there … when she is sitting there saying to you ‘I will be home next time you come’ I feel guilty you know, why can’t we provide that for you”. Similarly, Patricia spoke of internal difficulties related to caring for her father, expressing that “I remember dad always saying you know ‘I’ll look after you till death us do part’ and obviously we hadn’t been able to do that”, with Maggie sharing that “it’s a bit selfish because you think one mum could bring up three children, but three children couldn’t look after one mum when it comes to it … so you do feel guilty for that”.

Through participant reflections, it became clear that whilst many individuals experienced guilt when making the initial decision to move their relative into a care facility, many participants spoke of experiencing prolonged guilt in the knowledge they had made that decision and continued to experience guilt in the months and years following this transition. For Peter, he spoke of experiencing “guilt for a long time having to put him somewhere like that” whilst Gloria explained that “the worst feeling is guilt…you feel guilty…umm…you feel guilty about everything…you feel guilty about erm *starts to get tearful* … hang on a sec… that’s the worst part, the guilt”.

In addition, four participants spoke of their sadness in seeing their relative in an environment they had never anticipated they would be in, as detailed here by Josh: “yeah I mean it’s … it’s sad really, to see him in a home, because I don’t think I ever wanted to see him in a home, erm because he’s in this place where he doesn’t know anybody”.

#### “None of us knew anything about it”: fear and the unknown

A prominent theme that emerged within seven of the 13 interviews was the participants sense of apprehension and fear as they approached the transition of moving their relative into a care home. Many discussions centred on the lack of preparedness participants experienced, with some participants sharing how they received very little guidance or support from outside agencies regarding the logistics of this move. This had negative repercussions for many participants, contributing to fuelling further anxiety for what many participants recalled was a very stressful time already. As detailed by Malcolm, “it was, it was a challenge mainly because we had never done it before, that was the big challenge”. This view was shared by Mark, who expressed that “none of us knew anything about that, none of us had any experience of that, erm there was a definite fear of what that might cost, both emotionally and financially …”.

For many participants, the journey of moving their relative into a care home was the first time they had ever encountered this form of transition, with very few participants having previously spent time in a care facility and consequently having to rely on the media portrayals of care facilities or feedback from friends. As summarised here by Malcolm: “we had never been to a care home I didn’t know, we didn’t really have a comparison, I had never had any other relative in a care home so there was no expectation yeah so we didn’t know”.

As such, many participants spoke of the difficulty this caused in terms of choosing a care home for their relative, being unsure how to judge the quality of care provided by different facilities, with very little direction and it being “really hard knowing what to do” (Jessica) as “no one has had to go through it, it’s the first time you’ve gone through it” (Peter).

#### Relief and the release from worry: “a weight lifted”

Whilst many participants conveyed feelings of guilt and sadness, 10 of 13 participants spoke of *relief* after the transition, which was intimately connected with the appraisal of having done the right thing despite the arduous nature of the decision and transition. These 10 participants spoke of the relief they experienced once their relative was in a care facility, taking comfort in the knowledge that their relative was safe, subsequently living a life with less anxiety and worry, while also removing a burden from their own life.

Janet described a weight lifted in the following terms: “it was just that knowledge that when I went home I could breathe … I didn’t have to start everything, I wouldn’t be up half the night doing multiple washes, you know there’s so many little things it’s just … a big weight being taken off your shoulder.” Annie also implied disrupted sleep - she said “I knew I wasn’t going to get calls at 3 o’clock in the morning and all that stuff, erm so yeah that was a relief”.

Maggie also employed the metaphor of the weight being lifted, and focused more on the worry over her mother’s wellbeing, rather than the burden of looking after her. She said that “it was good for her and it was a bit of like a weight lifted for me and my sister cause we didn’t have to worry about where she was walking off to and things like that, we knew someone was looking after her and her medication, her food, it was all being done on a daily basis for her”.

In addition, the idea of stepping back a little and no longer being the first port of call for medical emergencies was frequently discussed. Peter referenced the positive aspect of release from worry after a long period: “erm … so yeah that was a positive thing, I never had the worry of having to go up and not being sure what I would find, you know I knew he was being cared for … so yeah thinking about it that took a lot of pressure off it all”.

### Reflective evaluation

#### “It was the right thing to do”: an improved quality of life for the older parent

Whilst many transitions were characterised by the emotions of guilt and sadness, another significant theme that emerged was the discussion surrounding the transition into a care facility being perceived as “the right thing to do”, mentioned by six participants, based on seeing how the care offered in the care home was, at long last, sufficient for the relative’s needs. Whilst participants reflected on a range of emotions that emerged throughout the transition, half of all participants spoke of their belief this was the best place for their relative, even though it was a difficult and emotional decision to make. As detailed by Patricia, “it was sad, there was guilt … um it was knowing she was in the best place, so you knew it was the right thing to do”. This belief was mirrored by Janet, who spoke of how the transition was full of “ups and downs all the way through” but was able to recognise the care home “were able to offer her a better quality of life than I was able to … with personal care and everything, because they knew … they’ve got the training you know, so it was worth it”.

Through these conversations, many participants discussed how although the transition was very challenging, they felt assured it was the right decision, with some participants reflecting that they could see their relative was “thriving” (Jennie) and that “it was right at that time for what she needed” (Maggie). In addition to this, some participants spoke confidently of their assurance that they had made the correct decision, with Jennie reflecting “I can put my hand on heart and say that I made the right decision and have no regrets” and Patrick commenting that “I am absolutely sure, absolutely sure it was the right decision … it was right but it wasn’t holy”.

#### “She was being looked after”: positive appraisals of the care environment

Over half of the interviews concluded with a period of reflective discussion on the impact of the transition. Seven of the 13 interviewees discussed the comfort they received knowing that their relative was receiving high quality care. Many participants spoke in detail about the differences they had noted since their relative was in the care facility, with this level of depth appearing to demonstrate how important this was to them. Maggie commented on the importance of their relative being well dressed, sharing “they would dress her lovely every day you know she never looked untidy, she never had any food stains down her … that meant the world to us to know she was being looked after …”. This was similar to Josh, who was able to recognise that “within the first few days it was noticeable how clean he was, so he’s always clean now, they cut his beard … his toenails have been cut, he smells clean which is a massive bonus”.

In addition to this, individuals appeared to take comfort in the knowledge their relative was being cared for, as detailed here by Mark: “the two of three occasions I visited I felt encouraged, I thought they were looking after my dad, you hear terrible stories but I thought that’s great, they’re looking after my dad” and Patrick: “it was very positive in that she was very well looked after there, I mean really well looked after”. This level of care appeared to support the individuals through the difficult elements of the transition, helping them to see the value of the move on the quality of life for their relative.

#### “The beginning of the end”: reflections on mortality during the transition

A further theme that emerged throughout the duration of the interviews related to that of participants slowly preparing for their relatives’ death. Six of the 13 participants discussed how they felt that moving their parent into a care facility symbolised the end of life, commenting that they knew the care home would be the last place their parent would live. Annie said that “it was the beginning of the end … which I think she still feels, I mean you end up in a home, it’s the last phase of your life”. Similarly, Maggie stated “the last place we moved her to which was the last place you would move someone to, you know it’s a nursing home … so we knew that was going to be her last home basically and that was probably the emotional side of it as that last move hit home for us because we knew where else is she going to go now, she is only going to get worse …”.

Linked to this visceral experience of mortality, some participants mentioned experiencing grief. Jenny, for example, stated that “I guess you start grieving for people before they died … I could see he declining so that’s the way it goes”. In summary, the transition to a care home is, for many children of an older parent, permeated with a sense of loss and grief, as the older parent moved into a residential environment that is likely to be the place they live until they die. The move to the care home means the ending of the parent’s life as an independent adult, which is also in itself an event that brings a sense of loss.

## Discussion

The overall purpose of this study was to examine the experiences of children of an older parent when supporting a move into a residential care or nursing facility. The study focused on the inner features of the transition that occurred alongside the recognised external features of moving from a personal residence to the care home. Thoughts, decision-making processes, emotions and holistic reflections that individuals reported were explored. This study provided evidence in support of Fisher’s (2012) model of life transitions and Schlossberg’s (2011) theoretical approach to coping with life transitions, which are discussed in reference to each meta-theme identified.

In terms of the *Decision Process* meta-theme, we found that the decision in all cases to be a complex negotiation between multiple stakeholders including the older family members and other stakeholders such as medical professionals. Just as found by Koenig et al. (2013), disagreement was an inherent part of the decision, and it compounded the stress of the situation relating to the frailty and difficulties of the older parent to the decision process being engaged in. The reflection that participants made about the decision itself was one that was considered a necessity rather than a voluntary issue. In so doing, this frames the decision as one that was not based on choice. It thus removes the matter of blame or causal attribution from them as key players in the decision. This may be an accurate framing of the event, and it may also be a way of cognitively appraising the situation in retrospect to protect the self from negative attribution (Speer & Delgado, 2020).

In terms of the *Conflicting Emotions* meta-theme, our findings supported the feature of Fisher’s model of transition that purports that, during a major life transition, individuals will experience a challenging range of diverse emotions, including anxiety, guilt, happiness and relief (Fisher, 2012). The findings also support previous research on the experience of carers of older adults in relation to the transition into care homes (Harrington et al., 2012). As with Fisher’s model, we found that some of these emotions overlap in reported occurrence, and that there is also a clear sequence, as anxiety and guilt give way to more positive emotions when the transition process concludes. Understanding the emotional impact of life transitions such as those disclosed in this research is important in light of the extensive evidence that transitions are challenging for mental health (Lee & Gramotnev, 2007). The interpersonal conflict, anxiety, guilt and sadness experienced through the care home transition may lead to mental health problems for many children of older adults. Fisher (2012) purports that this anxiety typically stems from an individual feeling out of control in the early stages of a transition or having inadequate information on how to adjust to the new environment; both of which were evident through the experiences shared within this study and outlined by other researchers within the field, such as Miller (2016).

In addition to the experience of anxiety, a further prominent feature was the overlapping emotions of guilt throughout the middle of the transitionary period. In this, whilst many participants spoke of their relief that their relative was receiving better quality care in a safe, secure environment, this relief was often offset by feelings of guilt, a finding supported in the literature by Harrington et al. (2012). While some individuals appeared to have experienced feelings of guilt temporarily, others appeared to be immersed within this emotion even after several years had passed.

It is important to highlight that some of these emotional experiences were particularly apparent for those children of older adults with a diagnosis of dementia or Alzheimer’s disease. This included the experience of guilt and shame regarding the decision, and fear and lack of knowledge regarding the care system, findings which were also supported by research from Afram et al. (2015) and Davison et al. (2019).

Schlossberg’s (2011) framework of coping with transition predicts a positive outcome through transition that relates to self, situation, support and strategies. The presence of social support through a transition is one feature that predicts a good outcome. This relates to our findings insofar, as social conflict, which is in many ways the opposite of social support, was found to be a major source of stress through the decision process. This conflict was for some participants with the older adult moving into the home and for others it was with other family members or stakeholders that disagreed with the decision per se, or with features of the decision such as which care home to move to. This finding also fits with previous research and signifies the importance of close personal relationships and networks when navigating a major life transition in relation to positive mental health and wellbeing (Ryan et al., 2012).

In terms of the Reflective Evaluation meta-theme, six out of 13 participants reflected positively on the transition as one that led to positive outcomes for their older parent. The other seven participants did not provide a clear view on the outcome. Four participants also discussed the deep sense of mortality that they felt during the transition. This relates to the finding that an elevated sense of mortality is common in all periods of life transition in later life (Robinson & Stell, 2015; Bridges, 2020).

## Practical implications

The results from this study have the potential to inform future practice surrounding support provided to individuals who are engaged in the process of deciding to move an older parent or relative into a care environment. Key findings could be utilised to produce accessible documentation which individuals could use to prepare themselves for the transition. Through outlining the stages one may experience at different points of the transition, this may serve a normalising purpose in relation to the emotions individuals may go through. For a process which many individuals find bewildering and complex, with very little guidance available relating to the practicalities of the transition, gaining an awareness of the psychological effects individuals may experience could be highly beneficial in aiding the transition. This documentation could be provided through many routes, including GPs, social workers, hospital staff, or public places such as libraries or community centres.

Given this finding, hospital staff and practitioners within social care or community settings could be encouraged to explore the long-term care options with family members at an early stage, to help prompt families to have these discussions which may subsequently help individuals feel greater prepared. In addition, for those relatives who are unable to contribute to the decision being made as a result of their cognitive frailty, individuals may take comfort in the knowledge that this transition had already been formerly discussed, which subsequently may help reduce feelings of guilt.

## Limitations and recommendations for further research

It is important to now consider the limitations of this study. A key limitation relates to the sample. The use of convenience sampling meant that all participants interviewed were from middle to high socio-economic groups in the UK and White British. Many of the participants discussed how they were in a fortunate position to be able to afford the financial repercussions involved in the transition into a residential care facility. However, this could have been different had other individuals been interviewed in relation to causing additional money-related stressors or anxiety; both of which may have affected how they coped with the transition.

In addition to this, the COVID-19 pandemic meant that all interviews were conducted virtually over video software, rather than face-to-face. This meant it was difficult to assess body language, and occassionally interviews felt formal and impersonal even after rapport had been established. In addition to this, a few interviews were faced with connectivity issues which resulted in occasional disrupted communication. However, despite these limitations, this approach enabled us to reach participants in locations that we wouldn’t have been able to physically access; thus, broadening the geographical breadth of the sample.

Finally, all interviews were conducted retrospectively, requiring participants to reflect on their past experience of the transition. This resulted in a reliance on the participants level of recall, with a possibility for recall bias. Whilst we cannot assure that this did not occur, questioning in chronological order, the use of probes and asking participants for further clarification would have minimised this risk (Tong et al., 2007). Additionally, the specifics of the transition, often being highly emotive in relation to sadness, stress and anxiety, meant that most participants provided in-depth comments, as for many it was a difficult transition which has since been engraved in their memory. This is supported by research which highlights that the events which occur during periods of heightened emotional arousal are more memorable than neutral events (Van Giezen et al., 2005; Anderson et al., 2006) and thus may be less susceptible to recall bias.

A potential limitation relates to the issues of voluntary-based recruitment for interviews. There is evidence to suggest that individuals who come forward for interview are likely to have a narrative to tell that has a positive ending, rather than those who experienced a broadly negative ending at the end of a transition. Therefore, the sample considered here may be biased towards positive outcomes from the transition of their parent to a care home.

Given the above limitation of retrospective narratives about the transition to care homes, future research within this field could be conducted in the form of a prospective longitudinal study. This would remove the reliance on reflection and follow individuals as they progress through this transition. Participants who are about to commence this transition could be contacted when they speculatively contact care homes for information and invited to take part, recruited through support agencies or via hospital admissions. This would provide the opportunity to conduct interviews or gather written data over a period of time. This has the benefit of obtaining a large volume of data for analysis and an assessment of how individuals differ in their experience of the transition whilst minimising problems related to impaired recall of data. Gender differences and cross-cultural differences should also be explored. Cultural differences are particularly important in light of the important contextual factors that relate to care home provision and decision-making in different cultures. Further research could also be conducted examining the difference between the transition for fathers and mothers going into care in later life, and the differences between sons and daughters. Finally, research that takes into account the state of physical health and neurological health prior to the transition would also be beneficial, given that those who are experiencing distress due to the older parent having dementia or other issues will affect the transition, for example through stronger feelings of relief and a stronger justification for institutional care. Finally, in order to further understand this complex transition, a more comprehensive examination of the role of distance and intensity of relationship between the individuals involved in the transition could be assessed. This could provide another layer of analysis and could help predict how an individual is likely to cope with the transition of moving their relative into a residential care facility.

## Data Availability

The data generated during and/or analysed during the current study are available from the corresponding author on request.
